# Development and validation of the Cystic Fibrosis Decisional Balance for Physical Activity scale (CF-DB-PA)

**DOI:** 10.1186/s12890-021-01471-0

**Published:** 2021-04-14

**Authors:** Valentine Filleul, Raphaëlle Ladune, Mathieu Gruet, Charlène Falzon, Amélie Fuchs, Laurent Mély, Meggy Hayotte, Jean-Marc Vallier, Philippe Giovannetti, Sophie Ramel, Anne Vuillemin, Karine Corrion, Fabienne d’Arripe-Longueville

**Affiliations:** 1 Université Côte-d’Azur, LAMHESS, Nice, France; 2 Université de Toulon, Unité de Recherche Impact de l’Activité Physique sur la Santé, Toulon, France; 3 Laboratoire de thérapeutiques non médicamenteuses innovantes Mooven, Montpellier, France; 4 Hôpital Renée Sabran, Centre de Ressources et de Compétences de la Mucoviscidose (CRCM), Giens, France; 5 Service de Pneumologie, Centre de Ressource et de Compétence de la Mucoviscidose (CRCM) adulte et pôle d’Activités Médicales Intersite de Médecine Physique et de Réadaptation APHM-CHU Hôpital Nord, Marseille, France; 6 Fondation Ildys, site de Perharidy, Roscoff, France

**Keywords:** Cystic Fibrosis, Physical activity, Exercise, Decisional balance, Barriers and facilitators, Questionnaire

## Abstract

**Background:**

People with cystic fibrosis (pwCF) derive several physiological and psychological benefits from regular physical activity (PA), but the practice is lower than recommended. Knowledge about the facilitators of and barriers to PA at the individual level is important to act positively on PA behaviors. This study validated the Cystic Fibrosis Decisional Balance for Physical Activity scale (CF-DB-PA) for adults with CF.

**Methods:**

French adults with CF were recruited in several specialist centres in France. The CF-DB-PA scale was validated following a quantitative study protocol comprising four stages: (1) tests of the clarity and relevance of a preliminary 44-item version and reduction analysis, (2) confirmatory factor analysis and tests of dimensionality through equation modelling analysis, (3) tests of reliability with Cronbach alphas for the internal consistency and a test–retest with a 2-to-3 week interval for temporal stability, and 4) tests of construct validity with Spearman correlations to measure the associations between each subscale and the theoretically related constructs (i.e., quality of life, PA and exercise tolerance).

**Results:**

A total of 201 French adults with CF participated in the validation study. The CF-DB-PA comprises 23 items divided into two factors: facilitators of and barriers to PA. Each factor is divided into three subscales: physical, psychological and environmental. The factors (facilitators and barriers) can be used independently or combined as a whole. A general score of decisional balance for PA can also be calculated. The bi-factor model presented satisfactory adjustment indexes: χ^2^ (194) = 362.33; *p* < .001; TLI = .87; CFI = .90; RMSEA = .067. The scale showed satisfactory internal consistency (Cronbach’s α = .77). The test–retest reliability was not significant for either subscale, indicating stability over time. The facilitators subscale correlated significantly with the self-reported score of PA (r = .33, *p* < .01) and quality of life (r = .24, *p* < .05). The barriers subscale correlated significantly with the self-reported scores of PA (r =  − .42, *p* > .01), quality of life (r =  − .44, *p* < .01), exercise tolerance (r =  − .34, *p* < .01) and spirometry tests (r =  − .30, *p* < .05).

**Conclusions:**

The CF-DB-PA is a reliable and valid questionnaire assessing the decisional balance for PA, the facilitators of and the barriers to PA for adults with CF in French-speaking samples.

## Introduction

Cystic Fibrosis (CF) is a recessive genetic disease that is life-shortening and affects multiple organs of the body. Currently, there is no cure, but advances in treatment offer pwCF greater life expectancy than in previous generations [1]. However, the treatments are complex and time consuming, involving daily medications, physiotherapy such as airway clearance, a strict diet, and physical activity (PA) [2]. PA, including sports, exercise, and recreational activities, is widely recommended as part of CF therapy due to its beneficial effects on physiological factors (e.g., improved anaerobic capacity) [3, 4], cardiovascular endurance [5], muscular strength [6], mucus clearance [7], psychological health (i.e., related to quality of life) [4], fatigue [8], and well-being [9].

Although the benefits of PA have been widely demonstrated, it appears that pwCF remain below the recommendations, with levels declining further throughout adolescence [10]. The literature reveals extensive explorations of the facilitators of and barriers to PA in children with CF, although only a few studies have focused on adults. However, with treatments improving, and according to recent annual data from the Cystic Fibrosis Foundation, more adults than children currently have CF, and a child born with CF today has a life expectancy of more than 45 years [11]. From a demographic perspective, the CF population is therefore older and is showing new comorbidities and specific adult constraints (e.g., more pwCF entering the workforce; a high prevalence of diabetes, which is not the case in children and could be a barrier to PA). For these reasons, a specific assessment of the facilitators of and barriers to PA in adults with CF is necessary. Also, the studies in the literature have only identified the facilitators and barriers at the group level in children (e.g., [12–14]), but no tool exists to identify them at the individual level in adults. Yet, clinically, this is essential for two main reasons: (a) time, as these people already have a heavy treatment load and thus may not be readily available for qualitative interviews, which can be lengthy and require expertise, and (b) the need for individualized care, as, given the wide genotypic and phenotypic variability in CF, pwCF are all very different and thus the facilitators of and barriers to PA are likely to vary from one individual to another.

In children with CF, fatigue, negative perceptions of PA, lack of motivation for and interest in PA, a perceived lack of PA, and lack of time, social support or available infrastructures appear to be the main barriers reported [10]. Concerning the facilitators in these patients, the same team of researchers identified the following: improved respiratory capacity and general health, positive perceptions of PA and peer and family social support [10]. A preliminary qualitative study conducted on adults with CF [15], highlighted relationships between physical barriers (i.e., fatigue, respiratory difficulties) and psychological barriers (i.e., lack of perceived physical ability; perceived risk of contamination), which were accentuated by environmental barriers (i.e., lack of time or social support). This study also identified: (a) physical benefits (e.g., improved respiratory capacity), (b) psychological benefits (e.g., well-being), and (c) social support from the environment and time-saving as the main facilitators of PA in adults with CF [15].

In order to successfully change the PA behavior of pwCF, it is necessary to identify and target its determinants. The Transtheoretical Model (TTM), initially created by Prochaska [16], is an integrative model conceptualizing intentional behavior change. The TTM is based on stages that explain when and how people might change their behavior. The Decisional Balance (DB), a key component of the TTM, reflects the relative weight of the pros and cons of changing for an individual. In order to assess the facilitators of (pros) and barriers to (cons) PA, Marcus et al. [17] developed the Decisional Balance Scale for Exercise (DBSE), which was later adapted in French by Eeckhout et al. [18]. This scale was developed for the general population and therefore was not designed to capture the specificities of vulnerable populations, especially the facilitators of and barriers to PA for adults with CF.

Therefore, the purpose of this study was to develop and to validate a DB scale to be used as a PA tool for adults with CF in French-speaking samples. This Cystic Fibrosis Decisional Balance for Physical Activity scale (CF-DB-PA) improves the assessment of the barriers to and facilitators of PA in adults with CF and, consequently, enhances the quality of the support than can be offered to help them adopt an active lifestyle.

## Materials and methods

### Procedure and participants

We developed and validated the scale through successive steps according to contemporary methodological recommendations [19, 20]: tests for clarity and relevance, dimensionality, reliability tests, and construct validity. Participants were recruited in several specialized CF centers in France. They were all adults with CF (age > 18 years). The exclusion criteria were people: (a) under guardianship or trusteeship; (b) in acute exacerbation phase; and (c) with cognitive impairment. The participants’ sociodemographic data are presented in Table 1. They were divided into four samples for the different steps of validation. The questionnaire was administered in paper form with relay persons (nurses and trainees) or online using LimeSurvey CE, version 2.06 + (LimeSurvey CE). This study was approved by the ethics committee from the French National Commission for Information Technology and Civil Liberties (CNIL T 39–2017) and all participants gave their informed consent before participation. All methods were carried out in accordance with relevant guidelines and regulations.

**Table 1:** Sociodemographic variables and clinical characteristics of study subjects. Descriptive statistics for each sample

Variable	Sample 1	Sample 2	Sample 3	Sample 4
	n = 9	n = 192	n = 73	n = 53
	Mean(SD)/N(%)	Mean(SD)/N(%)	Mean (SD)/N(%)	Mean (SD)/N(%)
Age (years)	–	33.0 (10.5)	30.1 (9.2)	32 (9.7)
Age at diagnosis (years)	–	6.1 (12.3)	3.1 (5.9)	3.5 (6.8)
Sex				
Female	5 (55.6)	111 (57.8)	38 (52.1)	28 (52.8)
Male	4 (44.4)	81 (42.2)	35 (47.9)	25 (47.2)
Further education* n (%)	–	153 (79.7)	52 (71.2)	40 (75.5)
Professional status n (%)				
Workers	–	117 (60.9)	40 (54.8)	25 (47.2)
Pensioners	–	4 (2.1)	0 (0)	0 (0)
Other people without activity and students	–	71 (37.0)	33 (45.2)	28 (52.8)
Weight (kg)	–	–	57.3 (11.6)	–
Height (m)	–	–	164.9 (9.1)	–
Body Mass Index (kg/m^2^)	–	–	20.9 (3.2)	–
Related disease (n, %)				
Diabete	–	52 (27.1)	25 (34.2)	20 (37.7)
Others	–	30 (15.6)	10 (13.7)	6 (11.3)
Distance 6MWT (meters)	–	–	607.2 (114.7)	–
FEV1 (%)	–	–	60.8 (23.7)	–
FEV1 (L)	–	–	2.1 (1.0)	–
FVC (%)	–	–	83.6 (21.2)	–

^*^Including university or high school

### Measures

#### CF-DB-PA

Items were developed based on existing scales in the general population (e.g., the Decisional Balance Scale for PA) [17], or other vulnerable populations (e.g., the Cancer Exercise Stereotypes Scale) [21] and on the results of a qualitative study of our research team [15]. The Wideband Delphi method, which entails successive rounds of expert input, was employed until consensus was reached [22]. A panel of nine experts was constituted (i.e., 5 researchers in Sport Sciences and Social Psychology, 1 nurse working with pwCF, 1 student in a Master’s program). The experts were identified according to (a) their previous research skills related to exercise in pwCF or to psychological factors of engagement in physical activity in vulnerable populations (5 researchers; 1 master student); (b) recommendations of a physician specialized in CF and part of our research team (2 physicians, 1 nurse). Initially, a list of 69 items was generated, including 40 items related to facilitators and 29 items related to barriers. After two rounds of the Delphi method, several items were deleted due to redundant or ambiguous features to CF. A preliminary version of the CF-DB-PA with 44 items was formulated, including 23 items related to facilitators and 21 items related to barriers. Items were divided into three subscales of facilitators: (a) physical (PHYF, n = 8), (b) psychological (PSYF, n = 5), (c) environmental (ENVF, n = 10); and three subscales of barriers: (a) physical (PHYB, n = 4), (b) psychological (PSYB, n = 7) and (c) environmental (ENVB, n = 10). Participants of sample 1 (see Table 1) were asked to answer questions on a 6-point Likert scale ranging from (1) " *Do not at all agree* " to (6) "*Totally agree*".

#### Quality of life

Quality of life (QOL) was assessed using the Cystic Fibrosis Questionnaire for patients over 14 years of age (CFQ14 +) [23]. This 49-item questionnaire is composed of nine QOL dimensions: physical functioning, energy/well-being, emotions, social limitations, role, embarrassment, body image, eating disturbances, and treatment burden. QOL was expected to be related to the CF-DB-PA dimensions in the convergent validity step.

#### Level of PA

Self-reported level of PA was assessed with the French version [24] of the Baecke Questionnaire [25], which has been recommended to assess PA in adults with CF [26]. This 16-item instrument with Likert-type responses ranging from 1 for “*Never*” to 5 for “*Always*”. The series of questions is divided in three habitual PA scores: occupational; physical exercise in leisure; and leisure and locomotion PA. The PA level was expected to be related to the CF-DB-PA dimensions in the convergent validity step.

#### Lung function and physical fitness

Forced Vital Capacity (FVC) and forced expiratory volume in one second (FEV_1_) were assessed by spirometry testing according to the ATS/ERS TASK FORCE recommendations [27]. Physical fitness was assessed with the six minute Walk Test (6MWT) [28]. The 6MWT was performed according to the recommendations of the ATS guideline [29]. Participants were instructed to walk back and forth in a 30-m length for 6 min. The total covered distance was recorded, rounded off to the nearest meter. These measures were expected to be related to the CF-DB-PA dimensions in the convergent validity step.

#### Sociodemographic

Sociodemographic information was requested of all participants at the end of the questionnaire and included gender, age, year of diagnosis, related diseases, education level and professional status. Also, lung function and physical fitness were evaluated.

### Statistical analyses

All statistical analyses were performed with SPSS (IBM Corporation, version 25) and AMOS (IBM Corporation, version 25) softwares.

### Assessment of clarity and relevance

Clarity and relevance were assessed by a large panel of CF professionals and specialists (n = 10; 1 physician, 5 nurses, 1 psychologist, 1 dietician, 1 physical therapist and 1 adapted PA professor) and pwCF (n = 9). They answered on a 6-point Likert scale, ranging from (1) “*Totally unclear*” to (6) “*Totally clear*” for the clarity test, and from (1) “*Completely irrelevant*” to (6) “*Highly relevant*” for the relevance test, and could comment on their answers. Problematic items were discussed between the members of the expert panel and modified until satisfactory scores were obtained.

### Tests of dimensionality

To test the dimensionality of the scale, we ran several structural equation modeling analyses [30]. Based in the recommendations [31], the fit indexes were: chi-square (χ^2^; significant values *p* ≤ 0.05), χ^2^ over degrees of liberty (significant values ≤ 3.00), Comparative Fit Index (CFI; value > 0.90), Tucker-Lewis fit index (TLI; value > 0.90), the root mean square error of approximation (RMSEA; value < 0.08) and the 90% confidence interval of RMSEA (ranging from 0.00 to 0.08).

### Tests of reliability

Internal consistency of each subscale was assessed using Cronbach’s alpha; a value > 0.70 is considered satisfactory and a value > 0.60 is considered marginally acceptable [32]. The test–retest reliability was calculated twice with a Student *t* test for paired samples on a reasonable interval of 2 to 3 weeks [33] and a minimum sample size of 50, as recommended [34]. Differences for *t* were considered statistically significant if *p* < 0.05.

### Tests of convergent validity

Spearman correlation coefficients were used to measure associations between the subscales of the CF-DB-PA and theoretically related constructs (i.e., QOL; PA and exercise tolerance).

## Results

### Study population

To conduct the successive stages of validation, we divided the participants into four samples: samples 1 (n = 9), 2 (n = 192), 3 (n = 73) and 4 (n = 53). Sample 1 was an independent group. Sample 3 was a subgroup of sample 2 and sample 4 was a subgroup of samples 2 and 3. Assessments of clarity and relevance were conducted on sample 1, tests of dimensionality were conducted on sample 2, test re-test reliability was conducted on sample 4, and the tests of construct validity were conducted on sample 3.

The global sample included 201 participants from ten CF centers of France (i.e., Nice, Giens, Roscoff, Montpellier, Grenoble, Marseille, Caen, Tours, Toulouse and Dunkerque).

### Tests of clarity and relevance

The clarity and relevance assessments of the preliminary 44-item version of the CF-DB-PA revealed an acceptable clarity score (M = 5.60, SD = 0.26) and an acceptable relevance score (M = 4.59, SD = 0.66) on a 6-point Likert scale. Three items obtained relevance scores below 3.00 and were removed. Six items with an ambiguous meaning were reworded. Then, redundant items were eliminated after discussion within the expert panel (n = 9) and after the item reduction analysis. Items with the higher clarity and relevance scores were saved, resulting in a new version with 23 items (see Table 2).

**Table 2:** Items of the 23-item version of the CF-DB-PA

	Category	Items
1	F_PHY_ 1.	Cela développe mes muscles respiratoires et réduit mon essoufflement. [It develops my respiratory muscles and reduces my shortness of breath.]
2	F_PHY_ 2.	Cela améliore mon endurance. [It improves my endurance.]
3	F_PHY_ 3.	Cela améliore ma force et ma masse musculaire. [It improves my strength and my muscle mass.]
4	F_PHY_ 4.	Une bonne condition physique favorise la réussite de la greffe. [A good physical condition promotes transplant success.]
5	F_PSYCH_ 1.	C’est l’occasion de penser à autre chose. [This is an opportunity to think about something else.]
6	F_PSYCH_ 2.	Cela me fait plaisir. [I am pleased to do it.]
7	F_PSYCH_ 3.	Cela me permet de rencontrer d’autres personnes. [It allows me to meet other people.]
8	F_ENVI_ 1.	Je bénéficie d’un encadrement compétent pour ma pratique. [I benefit from competent supervision for my PA.]
9	F_ENVI_ 2.	Je bénéficie de lieux adaptés à ma pratique. [I benefit from adapted places to do my PA.]
10	F_ENVI_ 3.	J’ai une offre de pratique près de chez moi. [I have a PA offer in my immediate area.]
11	B_PHY_ 1.	Cela me fatigue trop. [It fatigues me too much.]
12	B_PHY_ 2.	Je supporte mal l’effort physique. [I have trouble tolerating physical effort.]
13	B_PHY_ 3.	Je m’essouffle très vite. [I get short of breath really fast.]
14	B_PHY_ 4.	Je désature très vite. [I desaturate really fast.]
15	B_PSYCH_ 1.	J’ai peur d’être trop essoufflé.e. [I worry about getting short of breath.]
16	B_PSYCH_ 2.	J’ai peur d’être contaminé.e par des germes dans les lieux de pratique sportive. [I am afraid of being contaminated by germs in places for PA.]
17	B_PSYCH_ 3.	J’ai peur de tousser. [I am afraid of coughing.]
18	B_PSYCH_ 4.	J’ai peur d’être mal vu.e si je tousse devant les autres. [I am afraid of being frowned upon if I cough in front of others.]
19	B_PSYCH_ 5.	Je ne pense pas en être capable physiquement. [I don’t think I am physically able to do it.]
20	B_PSYCH_ 6.	Je n’arrive pas à suivre le rythme. [I can’t follow the rhythm.]
21	B_ENVI_ 1.	Je n’ai pas le temps à cause de mes contraintes familiales. [I don’t have time because of my family obligations.]
22	B_ENVI_ 2.	Je n’ai pas d’offre qui me convienne près de chez moi. [I don’t have a PA offer that works for me in my immediate area.]
23	B_ENVI_ 3.	Je n’ai pas l’encadrement adapté à mes besoins. [I don’t have supervision that is adapted to my needs.]

*PHYF* Physical facilitator; *PSYF* psychological facilitator; *ENVF* environmental facilitator; *PHYB* physical barrier; *PSYB* psychological barrier; *ENVB*: environmental barrier. For each item, participants responded on a 6-point Likert scale from (1) “Totally disagree” to (6) “Totally agree”. The introduction sentence was “The factors that would encourage me to regularly practice a physical activity are…” [*Les raisons qui m’inciteraient à pratiquer régulièrement une activité physique sont…*] for the facilitators and “The factors that would hold me back from regularly practicing a physical activity are…”, [*Les raisons qui me freineraient à pratiquer régulièrement une activité physique sont…*] for the barriers

### Tests of dimensionality

The first maximum likelihood confirmatory factor analysis (CFA) was conducted with the 23-item and six-factor model; four models were examined to assess the dimensionality of the scale [30]. The results of the model fit indexes are presented in Table 3.

**Table 3:** Fit indexes of the different models of the CFA (N = 192)

	χ^2^ (df)	*p*	RMSEA	CI of RMSEA 90%	TLI	CFI	Δχ^2^(df)	Δ*p*
Model a	955.89 (223)	< .001	.13	.12–.14	.52	.57		
Model b	715.44 (222)	< .001	.11	.10–.12	.67	.71	240.45 (1)	< .001
Model c	544.41 (227)	< .001	.09	.08–.10	.80	.82	171.03 (5)	< .001
Model d	362.33 (194)	< .001	.07	.06–.08	.87	.90	182.08 (33)	< .001

Model a: one-dimensional; Model b: first-order two-factor correlated; Model c: second-order hierarchical order; Model d: confirmatory bi-factor. χ^2^: Chi^2^; df: degrees of freedom; RMSEA: root mean square error of approximation; 90% CI: confidence interval of RMSEA 90, CFI: comparative fit index; TLI: Tucker-Lewis index of adjustment

**Fig. 1:**
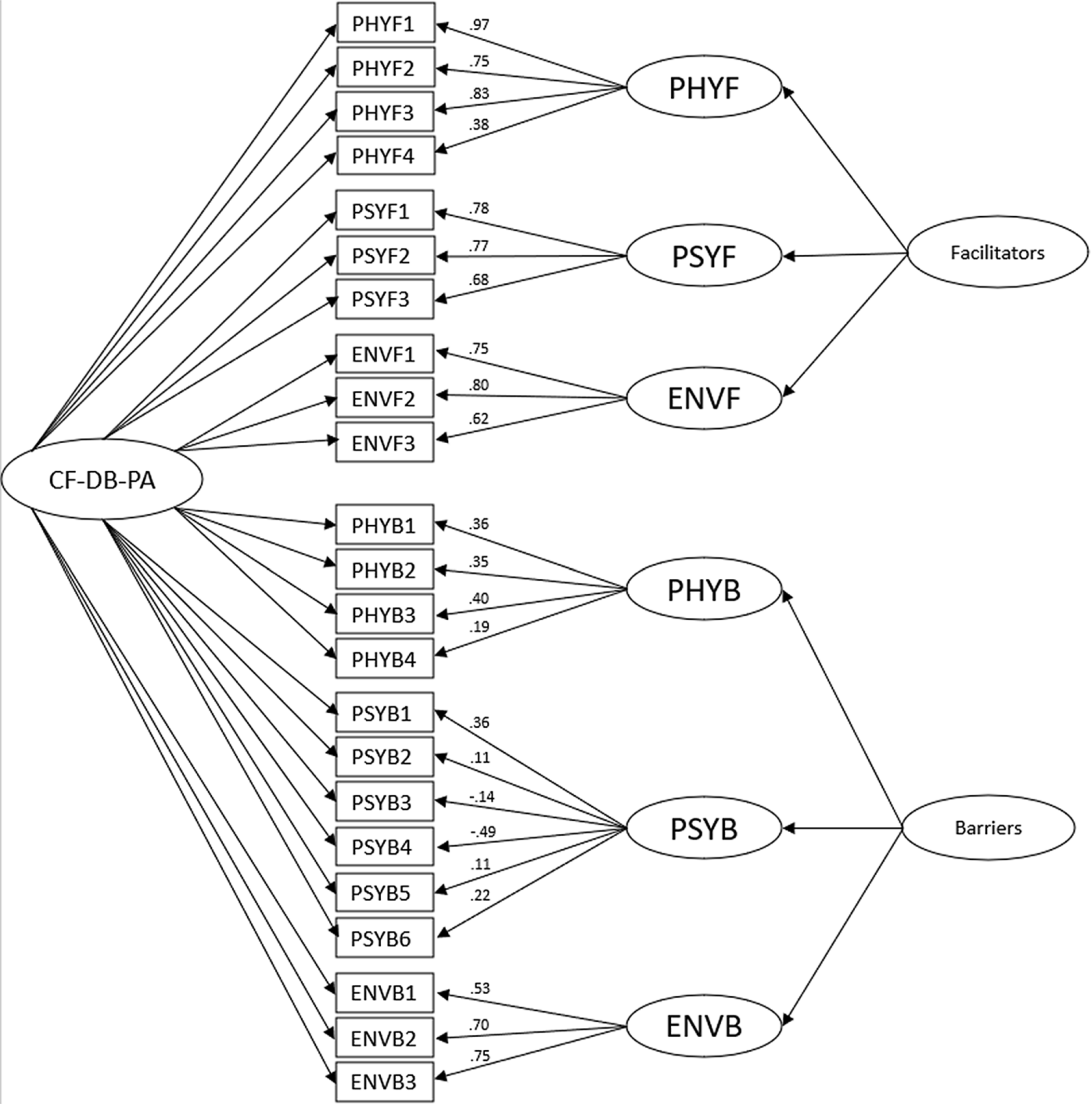
Estimation coefficients and standardized measurement errors of the confirmatory bi-factor model under testing. *Notes CF-DB-PA* cystic fibrosis decisional balance for physical activity scale; *PHYF* physical facilitator; *PSYF* psychological facilitator; *ENVF* environmental facilitator; *PHYB* physical barrier; *PSYB* psychological barrier; *ENVB* environmental barrier

Initially, the unidimensional model (model a) did not show satisfactory adjustment indexes. The following analysis examined a first-order model with two factors correlated (facilitators and barriers; model b) and a second-order hierarchical model (model c). At that time, adjustment indexes were not all satisfactory. Finally, analysis using a bi-factor model (model d) offered the most satisfactory adjustment indexes: χ^2^ (194) = 362.33; *p* < 0.001; TLI = 0.87; CFI = 0.90; RMSEA = 0.067; RMSEA LO/HI = 0.06/0.08 (Fig. 1).

### Tests of reliability

Cronbach alphas ranged from 0.65 to 0.88 (n = 192; i.e., α_Facilitators_ = 0.79; α_PHYF_ = 0.70; α_PSYF_ = 0.74; α_ENVF_ = 0.74; α_Barriers_ = 0.88; α_PHYB_ = 0.78; α_PSYB_ = 0.82; α_ENVB_ = 0.65) and 0.77 for the overall scale, demonstrating satisfactory internal consistency.

The test–retest reliability was not significant for either the barrier or facilitator scales, indicating stability over time for the questionnaire as a whole. At the subscale level, the Student *t* test was not significant for any subscales except the ENVF subscale. Table 4 presents the results of the *t* tests.

**Table 4:** Descriptive statistics for the test–retest reliability (n = 53)

	Time 1		Time 2		*t* tests
	M (SD)	α	M (SD)	α	
Facilitators	4.71 (.80)	.76	4.83 (.91)	.83	*t*(52) = − 1.70, *p* = .09
PHYF	5.23 (.61)	.60	5.23 (.61)	.61	*t*(52) = − .07, *p* = .95
PSYF	4.70 (1.22)	.74	4.78 (.1.28)	.72	*t*(52) = − .74, *p* = .46
ENVF	4.21 (1.13)	.42	4.48 (1.28)	.70	*t*(52) = − .2.2, *p* = .03
Barriers	2.76 (.90)	.86	2.68 (.96)	.88	*t*(52) = 1.18, *p* = .24
PHYB	3.06 (1.17)	.78	2.91 (1.12)	.76	*t*(52) = 1.75, *p* = .09
PSYB	2.63 (1.15)	.83	2.58 (1.14)	.82	*t*(52) = .48, *p* = .63
ENVB	2.58 (1.16)	.64	2.55 (1.19)	.62	*t*(52) = .31, *p* = .76

*PHYF* physical facilitator; *PSYF* psychological facilitator; *ENVF* environmental facilitator; *PHYB* physical barrier; *PSYB* psychological barrier; *ENVB* environmental barrier

### Tests of construct validity

Correlation analyses showed significant relationships in line with our expectations. Significant positive relationships were observed between the facilitators subscale and (a) the self-reported score of PA (r = 0.33, *p* < 0.01), and (b) the CFQ14 + (r = 0.24, *p* < 0.05). The barriers subscale was negatively related to (a) self-reported score of PA (r =  − 0.42, *p* < 0.01), (b) quality of life (r =  − 0.44, *p* < 0.01), (c) exercise tolerance (r =  − 0.34, *p* < 0.01) and (d) spirometry tests (r =  − 0.30, *p* < 0.05).

## Discussion

The purpose of this study was to develop and validate a scale to measure the Decisional Balance of PA tailored for a French-speaking adult CF population. Most of the studies focused on PA for pwCF have been conducted in children, and thus knowledge in adults remains scarce. Also, from a clinical point of view, the need for a fast and easy tool to assess PA modulators at an individual level in adults with CF emerged in parallel to the evolution of the CF population (e.g., longer life expectancy, bigger proportion of adults). The objective of this study was to fill this void. The development of the CF-DB-PA respected the stages of Vallerand’s [20] and Boateng’s [19] methodologies. The preliminary version was based on the findings of a qualitative study that identified the specific characteristics of facilitators of and barriers to PA in adults with CF [15], and the existing scales on the Decisional Balance for PA in the general population [18] and other vulnerable populations [21]. The CF-DB-PA comprises 23 items divided into six subscales: physical facilitators (4 items), psychological facilitators (3 items), environmental facilitators (3 items), physical barriers (4 items), psychological barriers (6 items), and environmental barriers (3 items). The dimensionality tests showed that the bi-factor confirmatory model had the best fit indexes. Thus, the three subscales of facilitators and the three subscales of barriers can be used independently or as a whole. In accordance with Eeckhout et al. [18], a general score of decisional balance for PA can be calculated.

The CF-DB-PA also demonstrated adequate-to-good internal consistency scores for all factors. There were no significant differences in the Student-*t* test over a 2- to 3-week interval for any subscale except ENVF. This difference can be explained by a change in the participant situation between T_1_ and T_2_ (e.g., in care at the CF center for the first time of measure and at home for the second). Thus, these results demonstrated the overall temporal stability of the CF-DB-PA. As predicted, several subscales of the instrument correlated in the expected direction with psychosocial measures such as quality of life from the CFQ14 + [23], PA levels from the Baecke questionnaire [24, 25] and exercise tolerance with the 6MWT [27] and spirometry tests [28], indicating strong convergent validity. Another strength of this scale validation comes from the representativeness of the sample, with participants recruited from several CF centers in France.

Some limitations must nevertheless be acknowledged. First, as for all rating scale, the self-reported nature of the responses may have been biased due to social desirability [35]. Also, this scale was validated for adults and may not be appropriate for children, younger adolescents, or their parents. Our samples did not allow testing for age and gender invariance. Complementary studies could thus be conducted to investigate invariance of this scale. The development of specific versions for children, adolescents and their parents would also be useful. Although the items are also presented in English in the table, the present validation only concerns the French-speaking samples. Future studies on the English validation of the tool or its translation into other languages would be interesting to internationalize its use and allow comparisons between countries.

## Conclusion

This study developed and validated the CF-DB-PA, which is the first measure assessing decisional balance for PA in adults with CF. The psychometric qualities of this scale were demonstrated. The CF-DB-PA offers new possibilities to better measure facilitators of and barriers to PA in adults with CF. The decisional balance score provides useful information on the patient’s stage of change and should help health professionals support and counsel adults with CF to better engage in PA. This scale may also contribute to the development of studies on the determinants of PA adherence in adults with CF. For future research and clinical practice, it might be useful to validate the scale in other languages and develop a digital version of this scale to obtain results more easily and quickly.

## Data Availability

The datasets used and/or analyzed during the current study are available from the corresponding author on reasonable request.

## Abbreviations

ATS: American Thoracic Society
CF: Cystic Fibrosis
CF-DB-PA: Cystic Fibrosis Decisional Balance for Physical Activity scale
CFA: Confirmatory factor analysis
CFI: Comparative Fit Index
CFQ14 +: Cystic Fibrosis Questionnaire for patients over 14 years of age
CNIL: Commission for Information Technology and Civil Liberties
DF: Degrees of freedom
ENVB: Environmental barriers
ENVF: Environmental facilitators
FEV1: Forced expiratory volume in one second
FVC: Forced vital capacity
M: Mean
PA: Physical activity
PHYB: Physical barriers
PHYF: Physical facilitators
PSYB: Psychological barriers
PSYF: Psychological facilitators
pwCF: People with Cystic Fibrosis
QOL: Quality of life
SD: Standard deviation
RMSEA: Root mean square error of approximation
TLI: Tucker–Lewis Index of adjustment
6MWT: Six minute Walk test
90% CI: Confidence interval of RMSEA 90, χ2: Chi^2^
